# A study to examine correlation between instructional leadership and teachers' motivation: evidence from TRNC

**DOI:** 10.3389/fpsyg.2026.1759126

**Published:** 2026-05-20

**Authors:** Hayat Kalkan, Ahmet Munir Acuner

**Affiliations:** Faculty of Educational Sciences, Cyprus Aydin University, Kyrenia, Cyprus

**Keywords:** correlation, instructional leadership role, teachers' motivation, teachers' motivation level, TRNC

## Abstract

The primary aim of the current study is to examine the correlation between the perceived instructional roles of TRNC school administrators and teachers' motivation levels. It should be emphasized that 450 teachers actively teaching at TRNC primary and secondary levels during the 2023–2024 academic year constituted the study sample. Results indicated that teachers perceived the instructional roles of their school administrators as “very high,” with identifying and sharing goals receiving the highest mean score. Similarly, teachers' motivation levels were high, except in the economic dimension. Findings indicated that the participating teachers showed statistically significant differences in age and level of education when evaluating their school administrators' instructional leadership roles. Furthermore, test results also suggested that participating teachers demonstrated statistically significant differences to their age and level of education. The Spearman correlation test results revealed a strong, positive, and significant relationship between the two concepts, indicating that instructional leadership roles had a significant positive relation on teachers' motivation levels.

## Introduction

The developments and changes in the economy and technology, especially in the 21st century, are thought to have necessitated nations reaching a certain level of knowledge and led to differences in individuals' needs. Therefore, it is considered essential for nations to adapt to these developments and changes and to use their resources in alignment with their objectives to sustain their existence (Aksoy, 2006).

Education plays a key role in the development of societies and in meeting their changing needs. Education is crucial in enhancing human resources' productivity and efficiency levels within organizations. Undoubtedly, productivity in educational institutions is also increases through individuals. More broadly, individuals' emotions, expectations, joys, and desires significantly improve productivity.

With changes in interests and needs, it is necessary to reconsider the goals of educational institutions and restructure them with stakeholder support (Yilmaz, 2010; p. 2, Zhang et al., 2025). In other words, it is impossible to describe a school that fails to meet societal expectations and needs as an “effective school.” For educational institutions to be effective, educational administrators and school staff need to be willing, dedicated, professional, motivated, and supportive of one another (Kurt, 2013; p. 1, Bellibaş et al., 2026). The concept of motivation is particularly significant in educational institutions. These institutions prepare individuals for “life” intending to achieve specific goals. (Çaliş 2012, p. 2) highlighted in his study that education brings to life individuals' hopes, ideals, and expectations, and that teachers, school administrators, and educational institutions play a substantial role in this process. The ability of educational institutions to achieve their predetermined educational goals, stay informed about educational developments, and function effectively is closely tied to the motivation of the teachers working within these institutions. Motivating teachers is one of the fundamental responsibilities of administration. (Çaliş 2012, p. 1) defined motivation as “the sum of hopes and beliefs that drive individuals and shape the direction of their actions.”

There is a strong relationship between teachers' motivation and their levels of job satisfaction and performance. The leadership qualities of school administrators play a significant role in motivating teachers. In educational institutions, teachers whose needs are met and whose expectations are fulfilled are more motivated and demonstrate greater dedication and more positive attitudes and behaviors in fulfilling their responsibilities and contributing to the institution's goals. However, teachers with low motivation will not exert the effort and dedication needed to achieve educational goals, potentially causing the institution they serve to stagnate in the long run (Ergen, 2009; p. 2, Elfira et al., 2024).

School administrators play a key role in motivating teachers. The “managerial roles” of school administrators have undergone significant changes. More broadly, it is now expected that school administrators recognize the importance of human needs, act in a way that aligns organizational goals with the staff's needs, adopt a philosophy of “continuous improvement” within their institutions, enhance the quality of teachers and education, and exhibit leadership qualities that increase teachers' motivation to teach (Balci, 2009; p. 4, Sariakin et al., 2025). (Kurt 2013, p. 3) emphasized in his research on the changing roles of school administrators that they need to influence employees in the institutions they serve and motivate educators in line with organizational goals. At this point, it becomes necessary for school administrators to act as “instructional leaders.”

The literature on instructional leadership provides insights into its definitions and characteristics. The focus of instructional leaders is on the processes of teaching and learning. (Altaş 2013, p. 20), highlighted that school administrators, as instructional leaders, should have the ability to inform educators about educational activities and motivate them to achieve educational goals. (Sişman 2004, p. 76) pointed out that school administrators, as instructional leaders, should determine specific goals and strategies, outline the school's mission and vision, and lead the sharing of these elements with the institution's stakeholders. (Inceler 2005, p. 25) emphasized that instructional leaders should possess sufficient knowledge and expertise to create a satisfactory and productive school climate for educators and stakeholders. Norbu and Lhabu (2021) define instructional leadership as actions taken by principals to improve teaching and learning. According to Norbu and Lhabu (2021), instructional leaders emphasize curriculum management and supervision of teaching and learning practices.

In general, instructional leaders strengthen collaboration among educators, establish the necessary educational resources and a climate for learning and teaching, and oversee and evaluate educators to ensure the effectiveness and quality of education. When the education system is considered as a whole, it is evident that the behaviors of instructional leaders significantly effect the quality of education and human resources in the institutions they serve, playing a crucial role in achieving the educational goals set by the institution. In other words, instructional leaders play a vital role in the realization of educational goals and the creation of an effective educational institution. Additionally, motivating teachers in this context is crucial. Examining these phenomena in relation to the correlation between the instructional leadership behaviors exhibited by school administrators and teacher motivation is essential.

## Literature review

### Instructional leadership

The literature presents numerous definitions of instructional leadership. Sucuoglu and Ulug (2022) defined the school leadership as a process that prepares teachers to address the changing needs of students and society. The school leader spends a significant portion of their time in instructional settings, striving to create a suitable learning climate and school culture through consultation with stakeholders (Sucuoglu and Ulug, 2022).

Alp et al. (2023) list the characteristics of an instructional leader as follows:

Possesses a vision: works to ensure all school stakeholders understand the school's goals and objectives.
Transform vision into action: prefers teamwork, emphasizing goals and expectations shared across the school.
Creates a supportive environment: establishes a school climate that enhances student achievement, motivates teachers, and aligns with objectives.
Is informed about all school activities: stays informed about school events and activities.
Mobilizes people with knowledge and skills: shares knowledge and experience, and when necessary, develops alternative teaching strategies to enhance teaching and learning activities.

### The concept of motivation

Employees are the primary source of power for an organization. Without employees, it is impossible for an organization to achieve its goals. While employees join organizations that fulfill certain needs of society, their primary aim is to meet their own needs. These needs can be material, psychological, and social. To the extent that an organization can meet these material and non-material needs, it can retain and engage its employees (Ergul, 2005).

Today, managers' success depends on their ability to unite the workforce under their management around the goals and objectives of the organization and ensure that they work toward these goals. Managers also aim to have employees reflect their knowledge, skills, and abilities in their contributions to the organization (Kantarcioglu, 2022).

(Koçel 2018) defines motivation as individuals taking initiative and acting in accordance with their desires to achieve a specific goal. (Taşdemir 2013) describes motivation as a source of life energy that begins with desire, drives individuals to act, ensures the continuity of action, focuses them on objectives, directs them toward positive outcomes, and helps them succeed.

Through motivation, employees can direct their efforts and actions toward the organization's goals. This not only increases organizational efficiency but also allows employees to find satisfaction in the work they do.

### Motivation in teachers

Schools are fundamental institutions that provide education and play a crucial role in shaping the future of nations, with humans being both their raw material and their product. Teachers are the most influential factor in helping schools achieve their objectives. In the teaching profession, which inherently requires sacrifice, it is essential to maintain teachers' willingness, enthusiasm, and passion. This necessitates, above all, a high level of motivation (Akman, 2017).

Understanding the factors influencing teacher motivation in the school environment is important for implementing measures to enhance it and enable teachers to carry out educational activities effectively. Additionally, understanding teachers' expectations from schools and stakeholders, and meeting these expectations, increases both their motivation and teaching performance (Karabag Köse et al., 2018).

In a master's thesis, Belle (2007) focused on the attitudes and behaviors of low-motivation teachers. Belle (2007) listed these attitudes and behaviors as follows:

- Frequently complain about their earnings and salaries.
- Continuously express dissatisfaction with the school environment.
- Follow instructions but avoid taking initiative.
- Demand clear definitions of their duties.
- Seek job security.
- Display limited interest and loyalty to their work.

Teacher motivation in schools holds significant importance in the following areas (Özdemir et al., 2014):

- Increasing commitment to the school and teaching profession,
- Adapting to innovation and change,
- Creating a positive school climate,
- Preparing a productive teaching environment for students,
- Enabling teachers to perform their duties with enthusiasm and contentment,
- Establishing a more peaceful educational environment,
- Achieving teaching objectives.

Guajardo (2011) identified factors affecting teacher motivation in developing countries as follows: workload and job difficulty, salary, incentives, appreciation and prestige, empowerment, career and development opportunities, participation in decision-making, educational materials, and activities.

### Characteristics of teachers with high and low motivation

In his research, Urhan (2018) discussed the characteristics of teachers with high and low motivation. According to the researcher, the characteristics of highly motivated teachers are as follows:

- They are eager to participate in activities organized by the educational institution they work for.
- They put in significant effort while performing their duties.
- They are highly active in both teaching and learning.
- They are closely engaged with their students and the subjects they teach.
- They have set clear goals related to themselves, their students, and their teaching.
- They have ideals.

The characteristics of teachers with low motivation are as follows:

- They lack a task-oriented mindset.
- They place little importance on the lessons they teach and the educational activities planned for those lessons.
- They are not effective in classroom management.
- They do not have clear goals regarding their school, lessons, or students.
- They fail to maintain the continuity of the teaching and learning process.

## Research methodology

### Research design and model

The current study had appointed quantitative research design. Therefore, findings will be presented as numerical values and interpreted in light of statistical analysis to examine the study's research questions (Karasar, 2016). Apart from this, as indicated earlier, the primary aim of the study is to explore the relationship between participating teachers' perceptions of their principals' instructional leadership roles and their motivation levels. Thus, the current study used a descriptive correlational model to examine the relationships between two or more variables (Çokluk et al., 2018).

### Population and sample of the study

The population of the current study consisted of 4,817 teachers teaching at the TRNC primary and secondary education levels during the 2023–2024 spring semesters. It can be inferred that the current study was planned for 450 teachers, based on the predicted sample table (95% confidence interval and 5% standard error), which was introduced by (Çingi 1994). In this study, a convenience sampling method was used, in which the researcher selected participants who were accessible and convenient for the study (Gravetter and Forzano, 2012).

Purposive and convenience sampling were specifically preferred by the researcher for their speed, accessibility, and cost-effectiveness. It is important that the sample size represents 10% of the population; the limitations of the sample include the inability to establish causal relationships and the lack of generalization to larger populations. Therefore, the research sample should be considered to have an exploratory and descriptive purpose.

### Aim of the research

The primary aim of the current research can be grouped under two main headings. The first of these is to evaluate primary and secondary school teachers who are actively teaching at TRNC public and private education institutions in terms of significant differences in line with socio-demographic variables (i.e., gender, age, salary, region, seniority at education institution and experience at teaching profession) in the context of instructional leadership roles which exhibited by school administrators and their motivation levels. Second, examine the correlational relationship between these two notions.

### Data collection tools

Data collection tools for the current research consisted of three parts: a personal information form, an instructional leadership scale, and, lastly, a teachers' motivation scale.

### Personal information form

The Personal Information form was designed to collect data on participants' socio-demographic characteristics. The form consisted of seven questions on gender, age, salary, teaching level, region of teaching, seniority at the educational institution, and experience in the teaching profession.

### Instructional leadership scale

The instructional leadership scale was launched by (Sişman 2004). The scale consisted of 50 items and five subdimensions, namely identifying and sharing school goals, managing the curriculum and teaching process, evaluating the teaching process and students, supporting the professional development of teachers, and creating a regular teaching and learning sphere. Participants are expected to articulate their responses to indicate the frequency of instructional leadership role for each item through five point Likert Scale that 1 = “Never”, 2 = “Very Rarely”, 3 = “Sometimes”, 4 = “Most of the Time”, 5 = “Always.” (Sişman 2004) tested the validity of the scale using factor analysis, and assessed its internal consistency and reliability through the Cronbach Alpha Coefficient test. Cronbach's Alpha was 0.92, indicating that the scale is highly reliable as a data-collection tool. This study reevaluates the Cronbach's alpha, which was found to be 0.988.

### Teachers' motivation scale

The teachers' motivation scale was proposed by Yildirim (2006). The scale is consisted from 30 items and three dimensions. Participating teachers expressed their responses through a 5-point Likert scale that 1 = not at all, 2 = Slightly, 3 = Moderate, 4 = A lot, 5 = Completely.

Yildirim (2006) tested the scale's validity using factor analysis and assessed its internal consistency and reliability using the Cronbach's alpha coefficient. Cronbach's Alpha was 0.945, indicating that the scale is highly reliable as a data-collection tool. This study reevaluates Cronbach's alpha, which was found to be 0.970.

### Findings

A total of 143 male participants (31.8%) and 307 female participants (68.2%) were enrolled in the study (Tables 1, 2).

**Table 1:** Socio-demographic profile of participating teachers.

Gender	*N*	(%)
Female	307	68.2
Male	143	31.8
Age		
21–30	56	12.4
31–40	161	35.8
41–50	157	34.9
At least 51 years old	76	16.9
Monthly income (TL)		
25,000–35,000	124	27.6
35,001–45,000	98	21.8
45,001–55,000	158	35.1
55,001 and above	70	15.6
Teaching region		
Nicosia	139	30.9
Famagusta	53	11.8
Kyrenia	61	13.6
Iskele	70	15.6
Lefke	127	28.2
Level of teaching employed		
Primary	111	24.7
Secondary	339	75.3
Seniority at education institution		
1–5 years	179	39.8
6–10 years	61	13.6
11–15 years	49	10.9
16–20 years	50	11.1
21 years and above	111	24.7
Working years		
1–5 years	99	22.0
6–10 years	68	15.1
11–15 years	62	13.8
16–20 years	68	15.1
21 years and above	153	34.0
Total	450	100

**Table 2:** Test of normality.

Dimensions	Kolmogorov–Smirnov				
	Statistic	*df*	Sig.	Skewness	Kurtosis
ISSG	0.143	450	0.000	1.263	2.145
MCTP	0.121	450	0.000	1.321	1.919
ETPS	0.120	450	0.000	1.278	1.211
SPDT	0.100	450	0.000	1.470	3.317
CRTLEC	0.130	450	0.000	1.517	3.677
Economic	0.092	450	0.000	1.667	2.701
Psychosocial	0.076	450	0.000	1.455	2.229
Organizational and administrative	0.076	450	0.000	1.317	2.213

ISSG, identifying and sharing school goals; MCTP, managing curriculum and teaching process; ETPS, evaluating teaching process and students; SPDT, supporting the professional development of the teachers, CRTLEC, creating a regular teaching and learning environment and climate.

Table 1 presents the socio-demographic characteristics of the participants. Of the 450 teachers, 68.2% were female and 31.8% were male. Regarding age, 12.4% were 21–30 years, 35.8% were 31–40 years, 34.9% were 41–50 years, and 16.9% were 51 years or older.

In terms of region of residence, 30.9% of participants were from Nicosia, 11.8% from Famagusta, 13.6% from Kyrenia, 15.6% from Iskele, and 28.2% from Lefke.

The majority of teachers (75.3%) were employed at the secondary level, while 24.7% worked in primary education.

Regarding institutional seniority, 39.8% had 1–5 years of experience, while 24.7% had 21 years or more. Similarly, 34.0% of participants had more than 21 years of total teaching experience.

The education level of the employed teachers was one of the variables in the current research. It can be stated that 24.7% of the teachers were teaching at the primary level, whereas 75.3% were in charge at the secondary level. When participants were viewed in terms of their seniority at the education institution; 39.8% of them had 1–5 years, 13.6% of them had 6–10 years, 10.9% of them had 11–15 years, 11.1% had 16–20 years and lastly 24.7% had minimum 21 years seniority at the education institution that they are currently teaching. Lastly when teachers monitored in terms of their working years, it was found that 22% of them were working for 1–5 years, 15.1% of them were working for 6–10 years, 13.8% of them were working as 11–15 years, 15.1% of them were working for 16–20 years and lastly 34% of them were working at least 21 years as a teacher.

Table 2 presents the results of the normality test for the two scales. Field (2009) had indicated that if *p*_sig_ is smaller than 0.05 it means that non-parametric statistical significant tests should be employed to test the hypothesis of the studies. However, skewness and kurtosis values also play a vital role in exploring whether the data shows a normal distribution or not. Cevahir (2020) in his book, stated that if the values of the skewness are + and greater than 1, it implies data is skewed to the right. Additionally, positive kurtosis values indicate the sharpness of the data. Therefore, the data gathered from the participants were not normally distributed but were right-skewed and had heavy tails; thus, non-parametric statistical tests should be employed for the present study. The normality of the data was assessed using the Kolmogorov–Smirnov test. As all *p*-values were below 0.05, the data did not meet the assumption of normality. In addition, skewness and kurtosis values were outside the acceptable range (±1), indicating non-normality. Therefore, non-parametric tests such as the Mann–Whitney *U*-Test and Kruskal–Wallis *H*-Test were employed.

The results of the Mann–Whitney *U*-Test indicated that there were no statistically significant differences between male and female teachers in terms of their perceptions of instructional leadership across all dimensions (*p* > 0.05). Although minor differences in mean rank scores were observed, these differences were not statistically meaningful. Similarly, no significant gender differences were found in teachers' motivation levels across economic, psychosocial, and organizational–administrative dimensions (*p* > 0.05) (Table 3).

**Table 3:** Mann–Whitney *U*-test results on perceived instructional leadership role score differences of participants'—gender.

Dimensions	Gender	*N*	MR	SR	*U*	*p*
ISSG	Female	307	226.43	69,513.00	21,666.000	0.824
	Male	143	223.51	31,962.00		
MCTP	Female	307	224.63	68,962.50	21,684.500	0.836
	Male	143	227.36	32,512.50		
ETPS	Female	307	223.37	68,574.00	21,296.000	0.610
	Male	143	230.08	32,901.00		
SPDT	Female	307	221.32	67,946.00	20,668.000	0.318
	Male	143	234.47	33,529.00		
CRTLEC	Female	307	221.98	68,148.00	20,870.00	0.391
	Male	143	233.06	33,327.00		

ISSG, identifying and sharing school goals; MCTP, managing curriculum and teaching process; ETPS, evaluating teaching process and students; SPDT, supporting the professional development of the teachers; CRTLEC, creating a regular teaching and learning environment and climate.

As shown in Table 4, participating teachers did not differ significantly (*p* > 0.05) in motivation level by gender.

**Table 4:** Mann–Whitney *U*-test Results on teachers' motivation level score differences of participants'—level of education employed.

Dimensions	Gender	*N*	MR	SR	*U*	*p*
Economic	Female	307	226.74	69,610.00	21,569.000	0.766
	Male	143	222.83	31,865.00		
Psychosocial	Female	307	225.54	69,242.00	21,937.000	0.992
	Male	143	225.41	32,233.00		
Organizationaland administrative	Female	307	223.54	68,626.00	21,348.000	0.639
	Male	143	229.71	32,849.00		

The Kruskal–Wallis *H*-test results indicated that the participating teachers differed significantly in their motivation levels by age (Table 5). The findings revealed statistically significant differences among age groups in both the psychosocial and organizational-administrative dimensions. To determine the sources of these differences, *Post hoc* pairwise comparisons were conducted using the Tamhane test. The Tamhane test results showed that Teachers aged 21–30 scored significantly higher than their colleagues aged 41–50 in the psychosocial dimension. Furthermore, teachers aged 21–30 also differed significantly from those aged 31–40, 41–50, and 51 and above on the organizational-administrative dimension. Overall, the findings suggest that teachers aged 21–30 tend to have higher motivation levels in the psychosocial dimension compared to their colleagues aged 41–50, and higher motivation levels in the organizational–administrative dimension compared to those aged 31–40, 41–50, and 51 and Tables 4, 6.

**Table 5:** Kruskal–Wallis *H*-test results on perceived instructional leadership role score differences of participants'—age.

Dimensions	Age	*N*	SO	SD	*X* ^2^	*p*
ISSG	21–30 (1)	56	272.09	3	10.812	**0.013**
	31–40 (2)	161	210.43			(1–2)
	41–50 (3)	157	217.67			**(1–3)**
	At least 51 (4)	76	239.28			
MCTP	21–30 (1)	56	283.49	3	14.801	**0.002**
	31–40 (2)	161	213.89			(1–2)
	41–50 (3)	157	211.48			**(1–3)**
	At least 51 (4)	76	236.32			
ETPS	21–30 (1)	56	277.39	3	11.402	**0.001**
	31–40 (2)	161	221.61			(1–2)
	41–50 (3)	157	209.81			**(1–3)**
	At least 51 (4)	76	227.93			
SPDT	21–30 (1)	56	269.41	3	8.534	0.36
	31–40 (2)	161	219.56			
	41–50 (3)	157	212.49			
	At least 51 (4)	76	232.61			
CRTLEC	21–30 (1)	56	266.51	3	7.904	0.46
	31–40 (2)	161	212.91			
	41–50 (3)	157	219.14			
	At least 51 (4)	76	235.08			

ISSG, identifying and sharing school goals; MCTP, managing curriculum and teaching process; ETPS, evaluating teaching process and students; SPDT, supporting the professional development of the teachers; CRTLEC, creating a regular teaching and learning environment and climate. Bold values are highlight statistically significant results.

**Table 6:** Kruskal–Wallis *H*-test results on teachers' motivation level score differences of participants'—age.

Dimensions	Age	*N*	SO	SD	*X* ^2^	*p*
Economic	21–30 (1)	56	269.30	3	9.148	0.27
	31–40 (2)	161	229.96			
	41–50 (3)	157	212.23			
	At least 51 (4)	76	211.19			
Psychosocial	21–30 (1)	56	286.13	3	15.741	**0.001**
	31–40 (2)	161	224.87			**(1–3)**
	41–50 (3)	157	206.09			
	At least 51 (4)	76	222.27			
Organizational and administrative	21–30 (1)	56	284.96			
	31–40 (2)	161	225.81	3	15.142	**0.002**
	41–50 (3)	157	206.67			(1–2)
	At least 51 (4)	76	219.93			(1–3), (1–4)

Bold values are highlight statistically significant results.

Table 7 portrays that participating teachers were statistically significant at evaluating their school administrators' leadership roles (*p* < 0.05). To be more specific it can be elicited that participating teachers who were teaching at primary school had evaluated the instructional roles of their school administrators more positive at all dimensions when compared with their colleagues who were teaching at secondary school (Table 7).

**Table 7:** Mann–Whitney *U*-test results on perceived instructional leadership role score differences of participants'—level of education employed.

Dimensions	Level of education employed	*N*	MR	SR	*U*	*p*
ISSG	Primary School	111	270.77	30,055.50	13,789.500	**0.000**
	Secondary School	339	210.68	71,419.50		
MCTP	Primary School	111	267.81	29,726.50	14,118.500	**0.000**
	Secondary School	339	211.65	71,748.50		
ETPS	Primary School	111	277.83	30,839.50	13,005.500	**0.000**
	Secondary School	339	208.36	70,635.50		
SPDT	Primary School	111	278.95	30,963.00	12,882.000	**0.000**
	Secondary School	339	208.00	70,512.00		
CRTLEC	Primary School	111	276.03	30,639.50	13,205.50	**0.002**
	Secondary School	339	208.95	70,835.50		

ISSG, identifying and sharing school goals; MCTP, managing curriculum and teaching process; ETPS, evaluating teaching process and students; SPDT, supporting the professional development of the teachers; CRTLEC, creating a regular teaching and learning environment and climate. Bold values are highlight statistically significant results.

Mann–Whitney *U*-test results indicated that teachers were statistically significantly different across all sub-dimensions (*p* < 0.05) in terms of their motivation level, depending on the level of education they employed. Results were interpreted using mean rank (MR) values, which indicated that teachers teaching at primary schools tend to have higher motivation than their colleagues at the secondary school level (Table 8).

**Table 8:** Mann–Whitney *U*-test results on teachers' motivation level score differences of participants'—level of education employed.

Dimensions	Level of education employed	*N*	MR	SR	*U*	*p*
Economic	Primary School	111	283.94	31,517.00	12,328.000	0.000
	Secondary School	339	206.37	69,958.00		
Psychosocial	Primary School	111	273.28	30,334.00	13,511.000	0.000
	Secondary School	339	209.86	71,141.00		
Organizational and administrative	Primary School	111	281.12	31,204.00	12,641.000	0.000
	Secondary School	339	207.29	70,271.00		

Kruskal–Wallis *H*-test results postulate that participating teachers were indifferent (*p* > 0.05) at evaluating their administrators' instructional leadership on context of working years (Table 9).

**Table 9:** Kruskal–Wallis *H*-test results on perceived instructional leadership role score differences of participants'—work experience.

Dimensions	Work experience	*N*	MR	SD	*X* ^2^	*p*
ISSG	1–5 years (1)	99	228.68	4	5.787	0.216
	6–10 years (2)	68	228.53			
	11–15 years (3)	62	194.51			
	16–20 years (4)	68	215.12			
	21 years and above (5)	153	239.27			
MCTP	1–5 years (1)	99	240.07	4	7.927	0.094
	6–10 years (2)	68	238.71			
	11–15 years (3)	62	197.19			
	16–20 years (4)	68	200.36			
	21 years and above (5)	153	232.85			
ETPS	1–5 years (1)	99	229.54	4	8.130	0.087
	6–10 years (2)	68	256.32			
	11–15 years (3)	62	208.98			
	16–20 years (4)	68	197.63			
	21 years and above (5)	153	228.27			
SPDT	1–5 years (1)	99	226.42	4	5.530	0.237
	6–10 years (2)	68	252.38			
	11–15 years (3)	62	206.08			
	16–20 years (4)	68	208.55			
	21 years and above (5)	153	228.36			
CRTLEC	1–5 years (1)	99	225.07	4	5.624	0.229
	6–10 years (2)	68	236.06			
	11–15 years (3)	62	199.76			
	16–20 years (4)	68	208.96			
	21 years and above (5)	153	238.87			

ISSG, identifying and sharing school goals; MCTP, managing curriculum and teaching process; ETPS, evaluating teaching process and students; SPDT, supporting the professional development of the teachers; CRTLEC, creating a regular teaching and learning environment and climate.

Table 10 illustrates the Kruskal–Wallis *H*-results which appointed to test statistical significance between participating teachers in terms of their motivation level on context of work experience. Findings stressed that participating teachers were not statistically significant from each other in terms of their motivation level on context of work experience (*p* > 0.05; Table 10).

**Table 10:** Kruskal–Wallis *H*-test results on teachers' motivation level score differences of participants'—work experience.

Dimensions	Work experience	*N*	MR	SD	*X^2^*	*p*
Economic	1–5 years (1)	99	231.09	4	4.137	0.388
	6–10 years (2)	68	247.99			
	11–15 years (3)	62	225.63			
	16–20 years (4)	68	227.25			
	21 years and above (5)	153	211.06			
Psychosocial	1–5 years (1)	99	237.78	4	7.349	0.119
	6–10 years (2)	68	257.05			
	11–15 years (3)	62	210.80			
	16–20 years (4)	68	211.32			
	21 years ans above (5)	153	215.79			
Organizational and administrative	1–5 years (1)	99	240.79	4	6.393	0.172
	6–10 years (2)	68	250.98			
	11–15 years (3)	62	219.65			
	16–20 years (4)	68	215.74			
	21 years ans above (5)	153	210.99			

Kruskal–Wallis *H*-test results signified that participating teachers were not statistically significant from each other (*p* > 0.05) at evaluating their administrators' instructional leadership on context of their seniority at education institution (Table 11).

**Table 11:** Kruskal–Wallis *H*-test results on perceived instructional leadership role score differences of participants'—seniority at education institution.

Dimensions	Seniority at education institution	*N*	SO	SD	*X* ^2^	*p*
ISSG	1–5 years (1)	179	226.29	4	5.374	0.251
	6–10 years (2)	61	198.38			
	11–15 years (3)	49	216.68			
	16–20 years (4)	50	253.45			
	At least 21 years (5)	111	230.43			
MCTP	1–5 years (1)	179	229.79	4	1.584	0.812
	6–10 years (2)	61	209.43			
	11–15 years (3)	49	215.71			
	16–20 years (4)	50	230.00			
	At least 21 years (5)	111	229.70			
ETPS	1–5 years (1)	179	221.20	4	0.396	0.983
	6–10 years (2)	61	231.69			
	11–15 years (3)	49	228.76			
	16–20 years (4)	50	225.37			
	At least 21 years (5)	111	227.65			
SPDT	1–5 years (1)	179	221.15	4	0.882	0.927
	6–10 years (2)	61	226.47			
	11–15 years (3)	49	228.50			
	16–20 years (4)	50	240.18			
	At least 21 years (5)	111	224.05			
CRTLEC	1–5 years (1)	179	222.40	4	4.047	0.400
	6–10 years (2)	61	200.04			
	11–15 years (3)	49	236.22			
	16–20 years (4)	50	244.14			
	At least 21 years (5)	111	231.36			

ISSG, identifying and sharing school goals; MCTP, managing curriculum and teaching process; ETPS, evaluating teaching process and students; SPDT, supporting the professional development of the teachers; CRTLEC, creating a regular teaching and learning environment and climate.

The results of the Kruskal–Wallis *H*-test indicated that teachers' seniority at the educational institution was not statistically significant in relation to their motivation levels (*p* > 0.05) (Table 12).

**Table 12:** Kruskal–Wallis *H*-test results on teachers' motivation level score differences of participants'—seniority at education institution.

Dimensions	Seniority at education institution	*N*	SO	SD	*X* ^2^	*p*
Economic	1–5 years (1)	179	224.01	4	7.615	**0.107**
	6–10 years (2)	61	223.96			
	11–15 years (3)	49	268.68			
	16–20 years (4)	50	229.92			
	At least 21 years (5)	111	207.69			
Psychosocial	1–5 years (1)	179	232.50	4	2.496	0.645
	6–10 years (2)	61	222.98			
	11–15 years (3)	49	239.89			
	16–20 years (4)	50	220.33			
	At least 21 years (5)	111	211.58			
Organizational and administrative	1–5 years (1)	179	233.51	4	4.718	0.317
	6–10 years (2)	61	216.37			
	11–15 years (3)	49	251.09			
	16–20 years (4)	50	218.60			
	At least 21 years (5)	111	209.41			

Bold values are highlight statistically significant results.

To test the correlation between instructional leadership and teachers'motivation level, the Spearman Rank Correlation Coefficient Test was employed. Spearman Rank Correlation Coefficient (*r*) revealed a significant, positive, and strong correlation (*r* = 0.696) between instructional leadership and teachers' motivation level (Table 13).

**Table 13:** Spearman rank correlation coefficient test result.

Instructional leadership roles and teachers' motivation level spearman rank correlation coefficient result	Instructional leadership
Instructional leadership	1
One-tailed relationship	**0.000**
Teacher motivation level	**0.696**

Bold values are highlight statistically significant results.

The results of the simple linear regression analysis indicated that instructional leadership had a positive and statistically significant value on teacher motivation (β = 32.684, *p* < 0.05). Furthermore, instructional leadership explained 48.4% of the variance in teacher motivation (*R*^2^= 0.484), indicating a substantial predictive relation (Table 14).

**Table 14:** Simple linear regression test result.

Dependent variable: teachers' motivation			
Independent variable	Beta (β)	*T*	*p*
Instructional leadership	32.684	8.209	**0.000**
***R***, *R*^2^ **vs**. ***F*** **values**	***R***	*R* ^2^	***F*** **value**
	0.696	0.484	420.371

Bold values are highlight statistically significant results.

## Discussion

The socio-demographic variables of the study included gender, age, monthly income, region of duty, education level, professional seniority, and years of service in the educational institution. According to the results of the normality test, the data did not meet the assumption of normality. Therefore, non-parametric methods such as the Mann–Whitney *U* and Kruskal–Wallis *H*-tests were employed to analyze the data for significant differences. The relationship between the instructional leadership roles of school principals and teacher motivation was examined using the Spearman Rank Correlation Coefficient. Additionally, the impact of school principals' instructional leadership roles on teacher motivation was assessed using simplea Linear linear regression analysis.

The participants' responses regarding the evaluation of school principals' instructional leadership roles did not significantly differ based on gender. Various studies have examined the relationship between gender and instructional leadership, but they have yielded conflicting results. Some studies found significant differences (Kaşal, 2021; Sümür, 2021), while others did not (Aksoy and Işık, 2008; Altaş, 2013; Yilmaz and Kurşun, 2015; Uysal and Bilgivar, 2023). The findings of this study support the results of Aksoy and Işık (2008), (Altaş 2013), Yilmaz and Kurşun (2015), and Uysal and Bilgivar (2023). The reason for this finding is that both male and female teachers share similar views when evaluating the instructional leadership roles of school principals, which makes gender an insignificant variable in determining perceived instructional leadership levels. On the other hand, studies investigating whether there is a significant difference between gender and motivation have also yielded different results. For example, Dil (2020) and Kurtar (2018) did not find a significant difference in motivation levels based on gender, while Ayaydin and ve Tok (2015), Çoban (2019), and Memişoglu and Kalay (2017) found a significant difference. This study, however, did not find a significant difference between gender and motivation levels. These findings are consistent with Dil (2020) and Kurtar (2018). The reason for this result is that the responses of participating teachers based on gender regarding motivation were similar.

This study revealed that participants' ages significantly influenced their evaluation of school principals' instructional leadership roles. Specifically, teachers aged 21–30 rated principals' instructional leadership roles more positively in the subdimensions of setting and sharing school goals, managing educational programs and teaching processes, and evaluating teaching processes and students, compared to teachers aged 31–40 and 41–50. Previous research by Okçu and Senyigit (2019) and Kaşal (2021) found a significant difference between age and instructional leadership roles, Yilmaz and Kurşun (2015) did not. This finding might be attributed to the fact that principals, in the subdimensions of setting and sharing school goals, managing educational programs, and evaluating teaching processes, provide more up-to-date information to teachers aged 21–30 than to their older counterparts.

Additionally, this study found significant differences in motivation levels based on participants' age. Teachers aged 21–30 had higher motivation levels in the psychosocial, organizational, and managerial subdimensions compared to their colleagues aged 31–40 and 41–50. There are few studies in the literature examining the relationship between age and motivation, and these studies have produced conflicting results. Some studies (e.g., Çoban, 2019) found a significant difference, while others (Kurtar, 2018; Dil, 2020) did not. The reason for this finding in the present study could be the role played by the psychosocial and organizational factors—such as opportunities for independent work, social participation, recognition of decisions by administrators, and opportunities to present views and suggestions—in increasing the motivation levels of teachers in the 21–30 age group. This finding can be interpreted through the lens of Self-Determination Theory, which posits that individuals are more motivated when their needs for autonomy, competence, and relatedness are satisfied. Younger teachers may be more receptive to leadership practices such as feedback, collaboration, and professional development opportunities, which support these psychological needs and enhance motivation. The results also revealed significant differences based on the level of education. Teachers in primary education evaluated their principals' instructional leadership more positively and reported higher motivation than those in secondary education. This finding may be explained through instructional leadership theory proposed by Philip Hallinger, which emphasizes the role of school leaders in shaping teaching and learning environments. In primary schools, principals may have more opportunities to engage directly with teachers and instructional processes, thereby fostering stronger perceptions of leadership and higher motivation (Hallinger and Wang, 2015; Hallinger et al., 2025a).

There are few studies examining whether there are significant differences between educational level and instructional leadership or motivation. For instance, Eker (2021) found a significant difference between the educational level and evaluation of instructional leadership roles. In contrast, Dil (2020) found a significant difference between district of assignment and motivation levels. Another researcher, Özgün (2018), in a master's thesis, examined teachers' perceptions of instructional leadership roles and motivation levels as a function of institution type. This study found significant differences in all subdimensions of both instructional leadership and motivation among participants. According to the findings, primary education teachers evaluated principals' instructional leadership roles more positively and reported higher motivation than their colleagues in secondary education. This result closely resembles (Özgün, 2018) findings (Hallinger et al., 2025b). The reason for this finding may be that school principals in primary education receive more support from deputy principals and responsible teachers when fulfilling their managerial duties, allowing them more time to develop their instructional leadership roles.

Furthermore, the higher motivation levels of teachers in primary education compared to those in secondary education might be explained by the fact that principals in primary education implement more effective policies in areas such as autonomy, teamwork, expressing ideas, in-service training, career development opportunities, creating a warm and supportive school climate, and providing necessary materials for creating an effective learning environment.

Various studies have examined whether there is a significant relationship between professional seniority and instructional leadership. These studies have yielded varying results. In other words, while some studies (Aksoy and Işık, 2008; Altaş, 2013; Yilmaz and Kurşun, 2015) found no significant difference between instructional leadership and professional seniority, others (Yigit and Kaya, 2020; Kaşal, 2021; Uysal and Bilgivar, 2023) did find a significant difference. The findings of this study align with those of (Aksoy and Işık 2008), (Altaş 2013), and (Yilmaz and Kurşun 2015).

On the other hand, various studies have examined whether there is a significant difference between professional seniority and motivation levels (Ayaydin and ve Tok, 2015; Memişoglu and Kalay, 2017; Kurtar, 2018; Çoban, 2019; Dil, 2020; Bush, 2025). These studies have produced contradictory results. In other words, Ayaydin and ve Tok (2015), Memişoglu and Kalay (2017), and Çoban (2019) found a significant difference between professional seniority and motivation levels, while Dil (2020) and Kurtar (2018) found no significant difference. The findings of this study are consistent with those of Dil (2020) and Kurtar (2018). The reason for this finding is that, in this study, teachers with different levels of professional seniority shared similar views on their perceptions and evaluations of school principals' instructional leadership roles and motivation levels. Instructional leadership and teacher motivation are closely interconnected. Effective school leaders can influence teachers' motivation by creating supportive environments, encouraging professional development, and fostering collaboration. In this sense, instructional leadership can be viewed as a key predictor of teacher motivation.

The last socio-demographic variable in the study is the number of years of service in the educational institution. There is a limited number of studies examining the relationship between years of service in the institution and both instructional leadership and teacher motivation. In this study, no significant difference was found between the evaluation of instructional leadership roles and years of service within the institution. Similarly, Okçu and Senyigit (2019) also found no significant difference between years of service in the institution and the evaluation of instructional leadership roles. Furthermore, no significant difference was found between years of service in the educational institution and teacher motivation in this study. In the literature review, while Çoban (2019) found a significant relationship between years of service at the institution and teacher motivation, Kurtar (2018) found no significant relationship. In this study, no significant difference was found between the number of years worked in the educational institution and either instructional leadership roles or teacher motivation. This is likely because the number of years worked in the institution is not an effective variable in evaluating instructional leadership roles and determining teacher motivation. Additionally, teachers who joined the educational institution at different times tend to share similar perspectives regarding the evaluation of school principals' instructional leadership roles and their motivation levels.

## Conclusion

According to the Mann–Whitney *U*-test results, there were no significant differences between male and female participant teachers in their evaluations of school principals' instructional leadership roles and their motivation levels. However, significant differences were found across age groups in these evaluations. In other words, teachers aged 21–30 evaluated the instructional leadership roles of school principals more positively in the sub-dimensions of School Goals, Educational and Psychological Support, and Social-Organizational Development than their colleagues aged 31–40 and 41–50. Moreover, teachers aged 21–30 had higher motivation levels in the psychosocial and organizational-administrative sub-dimensions than their colleagues aged 31–40 and 41–50.

In terms of the education level variable, participant teachers differed significantly across all sub-dimensions of both instructional leadership and motivation. The results showed that teachers working at the primary school level evaluated their principals' instructional leadership roles more positively and had higher motivation levels than their colleagues at the secondary school level. In the variables of years of service and professional seniority, participants did not differ significantly in terms of the instructional leadership exhibited by school principals or their motivation levels.

Since the data did not meet the assumptions of normal distribution, the Spearman Rank Correlation Coefficient test was applied to assess the relationship between the two concepts. The correlation test revealed a significant, strong, and positive relationship between the concepts. In other words, teachers' positive perceptions of their school principals' instructional leadership roles were associated with higher motivation levels. As a result of the Simple Linear Regression Analysis, it was found that the independent variable, instructional leadership, had a significant and positive effect on teacher motivation.

## Recommendations

### Specific recommendations based on findings

- Since teachers aged 21–30 evaluated school principals' instructional leadership significantly more positively than their colleagues aged 31–40 and 41–50 in the sub-dimensions of School Goals, Educational and Psychological Support, and Social-Organizational Development, principals should design career-stage-targeted leadership practices—such as structured mentoring programmes, shared decision-making opportunities, and tailored professional development—to strengthen mid-career and senior teachers' perceptions of instructional leadership support.
- Given that teachers aged 21–30 also reported higher motivation levels than their older colleagues in the psychosocial and organizational-administrative sub-dimensions, principals should proactively address the motivational decline observed among mid-career and senior teachers by providing psychosocial support, fostering collegial networks, ensuring a fair distribution of organizational tasks, and formally recognizing long-serving teachers' contributions.
- Because secondary-school teachers evaluated principals' instructional leadership and reported motivation levels significantly lower than primary-school teachers across all sub-dimensions, secondary-school principals in particular should intensify their instructional leadership practices—including regular classroom observations, constructive feedback on teaching methods, collaborative curriculum planning, and stronger engagement with teachers' professional and psychosocial needs—to narrow this gap.
- As the regression analysis demonstrated a significant and positive effect of instructional leadership on teacher motivation, educational authorities and school principals should prioritize the development of instructional leadership capacity—through principal training programmes, coaching, and performance feedback systems—as a direct and evidence-based lever for raising teacher motivation.
- Since no significant differences were detected by gender, years of service, or professional seniority, school-level motivation and leadership-development policies need not be differentiated on the basis of these variables, allowing principals and policymakers to concentrate resources on the dimensions where meaningful differences were identified—namely, age group and school level.

### General recommendations

- School principals should strengthen their roles in supporting and developing teachers by fostering positive relationships, identifying areas for improvement, and providing opportunities for professional development. Additionally, principals should ensure teachers' participation in decision-making processes and address any shortages in educational materials.
- Principals should appreciate teachers' efforts through formal recognition and organize meetings to share new knowledge gained from training programs. Monitoring the application of learned skills and providing support where needed are crucial for achieving educational goals.
- Principals are encouraged to adopt a lifelong learning mindset, allocate resources for teacher training, and periodically observe classrooms to provide constructive feedback on teaching methods and time management.
- To improve teacher motivation, principals should collaborate with the Ministry of Education to enhance social benefits and implement performance evaluation systems, including peer and student feedback. High-performing teachers should be rewarded to foster motivation and commitment.

## Recommendations for future researchers

- Future studies can use mixed-methods designs, incorporating semi-structured interviews, to identify underlying causes of low motivation and barriers to instructional leadership, offering more actionable insights.
- In addition to instructional leadership and teacher motivation, researchers can explore related variables like teacher self-efficacy, autonomy support, perceived job respect, and teacher commitment. Mediating variables, such as the role of self-efficacy in the relationship between leadership and motivation, can also be analyzed.

## Data Availability

Raw data supporting the findings of this article are available from the authors upon request.
